# Susceptibility trends of swine respiratory pathogens from 2019 to 2022 to antimicrobials commonly used in Spain

**DOI:** 10.1186/s40813-023-00341-x

**Published:** 2023-10-19

**Authors:** Anna Vilaró, Elena Novell, Vicens Enrique-Tarancon, Jordi Baliellas, Lorenzo Fraile

**Affiliations:** 1 Grup de Sanejament Porcí, Lleida, Spain; 2 Department of Animal Science, University of Lleida – Agrotecnio Center, Lleida, Spain

**Keywords:** Antibiotics, Resistance, Minimum inhibitory concentration, Trend susceptibility analysis

## Abstract

**Background:**

Antimicrobial resistance is one of the most important health challenges in humans and animals. Antibiotic susceptibility determination is used to select the most suitable drug to treat animals according to its success probability following the European legislation in force for these drugs. We have studied the antibiotic susceptibility pattern (ASP) of *Actinobacillus pleuropneumoniae* (APP) and *Pasteurella multocida* (PM) isolates, collected during the period 2019–2022 in Spain. ASP was measured by determining minimum inhibitory concentration using standardized laboratory methods and its temporal trend was determined by logistic regression analysis of non-susceptible/susceptible isolates using clinical breakpoints.

**Results:**

It was not observed any significant temporal trends for susceptibility of *Actinobacillus pleuropneumoniae* to ceftiofur, florfenicol, sulfamethoxazole/trimethoprim, tulathromycin and tildipirosin during the study period (p > 0.05). Contrarily, a significant temporal trend (p < 0.05) was observed for quinolones (enrofloxacin and marbofloxacin), tetracyclines (doxycycline and oxyteracycline), amoxicillin, tiamulin and tilmicosin. On the other hand, it was not observed any significant temporal trends for susceptibility of *Pasteurella multocida* to quinolones (enrofloxacin and marbofloxacin), amoxicillin, ceftiofur, florfenicol and macrolides (tildipirosin, tulathromycin and tilmicosin) during the study period (p > 0.05). Contrarily, a significant temporal trend (p < 0.05) was observed for tetracyclines (oxyteracycline), tiamulin and sulfamethoxazole/trimethoprim.

**Conclusions:**

In general terms, pig pathogens (APP and PM) involved in respiratory diseases analysed herein appeared to remain susceptible or tended to increase susceptibility to antimicrobials over the study period (2019–2022), but our data clearly showed a different pattern in the evolution of antimicrobial susceptibility for each combination of drug and microorganism. Our results highlight that the evolution of antimicrobial susceptibility must be studied in a case-by-case situation where generalization for drug families and bacteria is not possible even for bacteria located in the same ecological niche.

**Supplementary Information:**

The online version contains supplementary material available at 10.1186/s40813-023-00341-x.

## Background

Porcine Respiratory Disease Complex (PRDC) is a syndrome due to a combination of infectious and non-infectious factors. *Actinobacillus pleuropneumoniae* (APP), *Pasteurella multocida* (PM), *Mycoplasma hyopneumoniae* (MH), *Bordetella bronchiseptica* (BB) and *Glaesserella* (Haemophilus) *parasuis* (GP) are the most common bacterial agents involved and Porcine reproductive and respiratory syndrome virus (PRRSV), swine influenza virus (SIV) and porcine circovirus type 2 virus (PCV2) are the most prevalent viral agents [1–6]. On the other hand, many non-infectious predisposing factors are also involved in PRDC, such as poor environmental conditions, density, stressors, season of the year, genetic background, and production flow (all in-all out versus continuous flow) [7–9]. If preventive medicine programs, such as improving environmental conditions, decrease density and stressors, combined with vaccination against the major viral and bacterial infectious etiologic factors [10] are not in place or fail, the use of antimicrobials with therapeutic or metaphylactic purpose in pigs may be necessary to control the relevant pathogens involved in respiratory disorders [11–14], contributing to most of the pig antimicrobial consumption [12, 15].

Antimicrobial resistance (AMR) threatens the successful treatment of bacterial infections in humans and animals [16, 17]. The use of antibiotics (AB) is a driver in the increase of AMR in bacterial populations, even following guidelines for prudent use of AB [18, 19]. AMR bacterial genes from livestock has been increasingly investigated for its potential to transfer AMR to humans via direct contact, the environment and contaminated food [20–22]. Nevertheless, the extent of this transmission remains uncertain due to the enormous complexity of the AMR epidemiology involving animals, environment, and humans being particularly studied for *Escherichia coli* and third generation of cephalosporins [23–25] but policy makers, in the European Union (EU), have developed legislation to monitor and regulate exhaustively the antibiotic use in animals, with special focus on livestock [26–28]. The current EU legislation regarding antimicrobials [26] have focused special attention to restrict as much as possible the use of antibiotics and prioritize the use of some AB families versus others in animals following the recommendations addressed by the European Medicine Agency in 2019. Thus, last resource AB (quinolones, cephalosporins and polymyxins) should only be used when no other options belonging to less risky categories (C and D) for AMR are available to treat animals with the goal to decrease AMR burden in humans in the long run [29, 30]. There is the risk that this long-term reduction of AB consumption in veterinary medicine could seriously hamper the care of animals and generate severe welfare issues if animals are not treated with the right antimicrobial when it is really needed.

Studies have also showed an association between antimicrobial use (AMU) in animals and AMR in human pathogens with a zoonotic origin [31] that could be really a bidirectional animal-human association highlighting the relevance of one-health paradigm [22]. However, the global effect of these actions, regarding the reduction of AMR at the human-animal-environment interface, is still under investigation, and very few scientific studies have shown encouraging results, limited to some antibiotics such as colistin [32, 33]. Thus, AMU is one key driver for AMR but other socio-economic factors should be also taken into account as drivers in AMR epidemiology as recently assessed [22]. Up to date, most of the AMR long-term monitoring data available are only from healthy animals that may not reflect the situation in veterinary bacterial pathogens [34]. At European level, the European Food Safety Authority (EFSA) coordinates a mandatory active monitoring of AMR in zoonotic (*Salmonella spp* and *Campylobacter spp*), indicator bacteria (*Escherichia coli*) and extended-spectrum-cephalosporin-resistant and carbapenemase-producing *E. coli* from healthy food-producing animals (cattle, poultry, pigs) at slaughter and meat following European directives [35, 36]. On the other hand, a coordinated and harmonized strategy for AMR monitoring for animal pathogens has just started at European level [37] to fill the gap for AMR data in pathogens from diseased animals. Thus, updated information will be generated to guide antimicrobial stewardship initiatives such as treatment guidelines, and to guide policymakers in regulating veterinary antibiotic use [38].

An important aspect of dealing with the AMR crisis is monitoring [39], which provides susceptibility data allowing to take action more effectively when necessary. Another goal of AMR monitoring is to analyse the temporal trends of AMR patterns for early warning of potential threats and decipher the impact of policies in animals regarding the use of AB in the long term. Thus, it is necessary to decipher the temporal susceptibility pattern of veterinary bacterial pathogens in livestock. Until now, there was a scarce of knowledge on the antibiotic susceptibility profiles of veterinary bacterial pathogens in Europe due to a lack of coordinated strategy between member states [38]. In this study, we present antibiotic susceptibility patterns for some of the most important pig respiratory pathogenic bacteria, collected during the period 2019–2022 in Spain.

## Results

### Bacteria isolation

From January 2019 to 2022, 1,827 samples were received from isowean, wean-to-finish and fattening farms suffering from clinical respiratory disease associated with PRDC from the main pig producing areas in Spain (Cataluña, Aragón, Murcia, Comunidad Valenciana and Castilla-León). The included farms belonged to most of the pig integration companies operating in each region. Only one isolate was included by farm across the study to avoid redundancy and overrepresentation of bacterial clones. In the case of sow farms, the samples were obtained from their nursery facility. Bacterial isolation for respiratory pathogens (APP, PM, and BB) was successful in 80% (1,461/1,827) of the cases, furthermore in 20% of the samples, more than one bacterial species was isolated. APP and PM MIC data were included in former analysis because, at least, 100 isolates were available for each year. Unfortunately, the number of BB isolates was too low to meet our research goals (between 21 and 53 isolates by year).

### Distribution of MIC by antimicrobial and microorganism across the years

MIC distributions (MIC minimum and maximum value observed (range), MIC_50_ and MIC_90_) and percentage of susceptible isolates are showed in Tables 1 and 2 for APP and PM, respectively.

**Table 1:** Minimum inhibitory concentration (MIC) distribution values of *Actinobacillus pleuropneumoniae* to quinolones (enrofloxacin and marbofloxacin), tetracyclines (doxycycline and oxytetracycline), beta-lactams (ceftiofur and amoxicillin), phenicols (florfenicol), sulfonamides (Sulfamethoxazol/trimethoprim), pleuromutilins (tiamulin) and macrolides (tilmicosin, tildipirosin and tulathromycin) from 2019 to 2022 in Spain

Year	Number of isolates	MIC range (µg/mL)	MIC_50_ (µg/mL)	MIC_90_ (µg/mL)	% susceptible isolates
*Enrofloxacin*					
2019	123	0.03-4	0.06	1	69.9
2020	195	0.03-4	0.06	0.5	86.5
2021	237	0.03-4	0.06	0.5	88.6
2022	228	0.03-4	0.06	0.5	84.7
Marbofloxacin					
2019	123	0.03-4	0.06	1	70.5
2020	195	0.03-4	0.03	0.5	86.2
2021	237	0.03-2	0.03	0.25	90.7
2022	228	0.03-4	0.03	0.25	92.9
*Doxycycline*					
2019	123	0.12-8	2	4	32.5
2020	195	0.5–16	2	4	19.5
2021	237	0.5–16	2	4	8.9
2022	228	0.5–16	2	4	12.3
*Oxytetracycline*					
2019	123	0.12-8	8	8	31.2
2020	195	0.5-8	8	8	22.6
2021	237	0.5-8	8	8	15.7
2022	228	0.25-8	8	8	23.9
*Ceftiofur*					
2019	123	0.06–0.12	0.06	0.06	100
2020	195	0.06–0.12	0.06	0.06	100
2021	237	0.06–0.25	0.06	0.06	100
2022	228	0.06–0.25	0.06	0.06	100
*Amoxicillin*					
2019	123	0.06-16	0,25	16	74.8
2020	195	0.12-8	0.5	8	66.7
2021	237	0.25-8	0.5	8	62.1
2022	228	0.12-8	0.5	8	74.6
*Florfenicol*					
2019	123	0.06-2	0.25	0.25	100
2020	195	0.25-8	0.25	0.50	98
2021	237	0.25-8	0.50	0.50	98.7
2022	228	0.25-8	0.50	0.50	99.1
*Sulfamethoxazol/trimethoprim*					
2019	123	0.06-8	0.12	4	89.4
2020	195	0.06-8	0.06	1	92.8
2021	237	0.06-4	0.12	1	95.4
2022	228	0.06-4	0.06	2	95.2
*Tiamulin*					
2019	123	2–32	16	16	99.2
2020	195	4–32	16	32	88.2
2021	237	4–32	16	16	98.3
2022	228	4–32	16	16	97.4
*Tilmicosin*					
2019	123	4–64	16	16	99.2
2020	195	4–32	16	32	88.7
2021	237	4–64	16	16	97
2022	228	4–32	16	16	90.3
*Tildipirosin*					
2019	123	2–64	4	8	99.2
2020	195	2–32	8	16	99.4
2021	237	2–16	8	8	100
2022	228	4–32	8	8	99.5
*Tulathromycin*					
2019	123	8–64	32	64	98.3
2020	195	16–64	32	64	100
2021	237	16–64	32	64	100
2022	228	16–64	32	64	99.1

MIC range is the minimum and maximum MIC value observed

**Table 2:** Minimum inhibitory concentration (MIC) distribution values of *Pasteurella multocida* to quinolones (enrofloxacin and marbofloxacin), tetracyclines (doxycycline and oxytetracycline), beta-lactams (ceftiofur and amoxicillin), phenicols (florfenicol), sulfonamides (sulfamethoxazol/trimethoprim), pleuromutilins (tiamulin) and macrolides (tilmicosin, tildipirosin and tulathromycin) from 2019 to 2022 in Spain

Year	Number of isolates	MIC range (µg/mL)	MIC_50_ (µg/mL)	MIC_90_ (µg/mL)	% susceptible isolates
*Enrofloxacin*					
2019	111	0.03–0.5	0.03	0.12	98.2
2020	100	0.03–0.5	0.03	0.12	97.8
2021	147	0.03-4	0.03	0.12	95.8
2022	178	0.03-4	0.03	0.06	97.1
*Marbofloxacin*					
2019	111	0.03–0.5	0.03	0.12	97.3
2020	100	0.03–0.5	0.03	0.12	98.9
2021	147	0.03-4	0.03	0.12	95.8
2022	178	0.03-4	0.03	0.12	97.1
*Doxycycline*					
2019	111	0,12 − 8	0.5	2	49.6
2020	100	0,12 − 8	0.5	2	50.6
2021	147	0,25 − 16	0.5	8	59.7
2022	178	0,25 − 16	0.5	2	62.6
*Oxytetracycline*					
2019	111	0,12 − 8	1	8	39.6
2020	100	0,25 − 8	1	8	33.3
2021	147	0,25 − 8	1	8	43.8
2022	178	0,12 − 8	0,5	8	61.7
*Ceftiofur*					
2019	111	0.06–0.25	0.06	0.12	100
2020	100	0.06–0.5	0.06	0.12	100
2021	147	0.06-1	0.06	0.25	100
2022	178	0.06–0.5	0.06	0.06	100
*Amoxicillin*					
2019	111	0.12-8	0.25	0.5	95.5
2020	100	0.12-8	0.25	0.5	92.3
2021	147	0.12-8	0.5	0.5	94.4
2022	178	0.12-8	0.25	0.5	95.3
*Florfenicol*					
2019	111	0.12-2	0.5	0.5	100
2020	100	0.25-8	0.5	0.5	97.8
2021	147	0.25-8	0.5	0.5	99.3
2022	178	0.25-8	0.5	0.5	98.8
*Sulfamethoxazol/trimethoprim*					
2019	111	0.06-8	0.12	4	80.2
2020	100	0.03-4	0.06	2	94.5
2021	147	0.06-4	0.06	2	90.3
2022	178	0.06-8	0.06	1	91.2
*Tiamulin*					
2019	111	2–64	16	32	62.2
2020	100	8–64	16	32	52.8
2021	147	8–64	32	32	39.6
2022	178	0.5–64	16	32	58.5
*Tilmicosin*					
2019	111	1–64	8	16	93.7
2020	100	2–64	8	16	94.5
2021	147	2–64	8	16	93.1
2022	178	1–32	8	16	95.3
*Tildipirosin*					
2019	111	0.5–64	1	2	97.3
2020	100	0.5-8	2	4	97.8
2021	147	0.5–64	2	4	95.8
2022	178	0.5-4	1	2	100
*Tulathromycin*					
2019	111	0.5–64	2	4	99.1
2020	100	1–8	2	4	100
2021	147	0.5–64	4	8	96.6
2022	178	0.5–64	2	4	96.3

MIC range is the mínimum and màximum MIC value observed

The APP isolates were highly susceptible (> 85%) to macrolides (tildipirosin, tulathromycin and tilmicosin), tiamulin, florfenicol, sulfamethoxazole/trimethoprim and ceftiofur across the study period (2019–2022). However, the antimicrobial susceptibility was intermediate (between 62 and 74.8%) for amoxicillin, low (between 8.9 and 32.5%) for tetracyclines and variable for quinolones (69.9 to 92.9%) during the study period (Table 1). *Pasteurella multocida* isolates, in contrast, showed high susceptibility (> 85%) to macrolides (tildipirosin, tulathromycin and tilmicosin), florfenicol, quinolones, amoxicillin and ceftiofur. However, PM antimicrobial susceptibility was intermediate and variable (33.3–62.6%) for tetracyclines and variable for sulfamethoxazole/trimethoprim (80.2–94.5%) and tiamulin (39.6–62.2%) during the study period (Table 2).

### Logistic model for *Actinobacillus pleuropneumoniae*

It was not observed any significant temporal trends for susceptibility to ceftiofur, florfenicol, sulfamethoxazole/trimethoprim, tulathromycin and tildipirosin during the study period (p > 0.05). Contrarily, a significant temporal trend (p < 0.05) was observed for quinolones (enrofloxacin and marbofloxacin), tetracyclines (doxycycline and oxytetracycline), amoxicillin, tiamulin and tilmicosin.

**Fig. 1:**
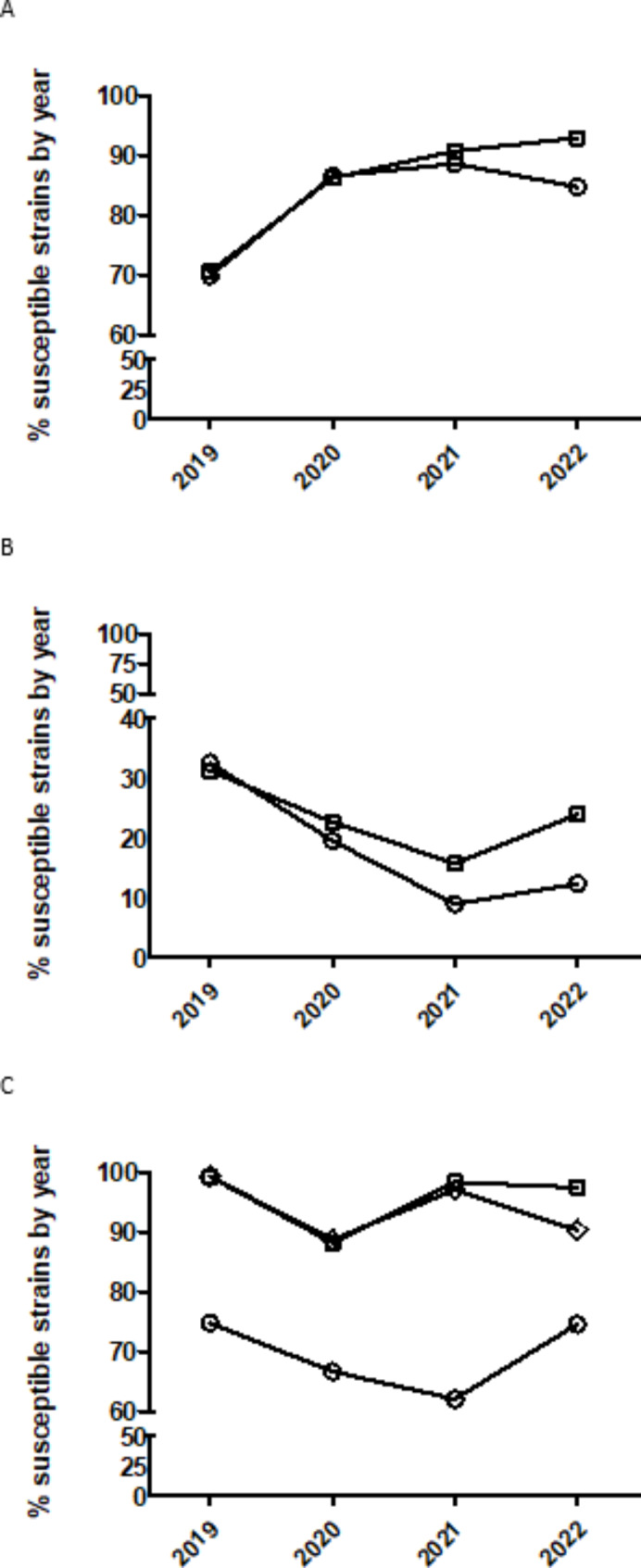
Percentage of susceptible isolates of *Actinobacillus pleuropneumoniae* by year for quinolones (enrofloxacin (circle) and marbofloxacin (square) (**A**)), tetracyclines (doxycycline (circle) and oxytetracycline (square) (**B**)) and amoxicillin (circle), tiamulin (square) and tilmicosin (diamond) (**C**)

In the case of quinolones, isolates from 2020 had significantly increased odds of being more susceptible than isolates from 2019 (Table 3; Fig. 1) remaining without significant changes later. For tetracyclines, the temporal trend is different between members of this family. Thus, for doxycycline, isolates from 2020 to 2021 had significantly decreased odds of being more susceptible than isolates from 2019 to 2020, respectively (Table 3; Fig. 1) remaining without significant changes in the year 2022 whereas, for oxytetracycline, the temporal trend was similar to doxycycline for the year 2020 and 2021 but only with a statistical (p < 0.1) tendency (Table 3; Fig. 1) but isolates from 2022 had significantly increased odds of being more susceptible than isolates from 2021 (Table 3; Fig. 1). In the case of amoxicillin, only isolates from 2022 had significantly increased odds of being more susceptible than isolates from 2021 (Table 3; Fig. 1). Finally, as for tiamulin and tilmicosin, the temporal trend was quite similar between both drugs during the year 2020 and 2021 (Fig. 1). Thus, isolates from 2020 to 2021 had significantly decreased and increased odds of being more susceptible than isolates from 2019 to 2020, respectively (Table 3; Fig. 1) and, only for tilmicosin, isolates from 2022 had decreased odds of being more susceptible that isolates from 2021 (Table 3).

**Table 3:** The adjusted odds ratio (95% confidence interval) describing the annual variation in susceptibility of *Actinobacillus pleuropneumoniae* (APP) isolates to quinolones (enrofloxacin and marbofloxacin), tetracyclines (doxycycline and oxytetracycline), beta-lactams (ceftiofur and amoxicillin), phenicols (florfenicol), sulfonamides (sulfamethoxazol/trimethoprim), pleuromutilins (tiamulin) and macrolides (tilmicosin, tulathromycin and tildipirosin) using the logistic regression model

Drug	Enrofloxacin	Marbofloxacin	Doxycycline	Oxytetracycline
Year	S (P = 0.0002)	S (P < 0.0001)	S (p < 0.0001)	S (p = 0.008)
20 vs. 19	S- 2.7 (1.6–4.8)	S- 2.6 (1.5–4.6)	S- 0.5 (0.29–0.84)	T (p = 0.09)
21 vs. 20	NS	NS	S- 0.4 (0.22–0.71)	T (p = 0.07)
22 vs. 21	NS	NS	NS	S- 1.69 (1.10–2.71)
**Drug**	**Ceftiofur**	**Amoxicillin**	**Florfenicol**	**Sulfamethoxazol/trimethoprim**
Year	NS	S (p = 0.01)	NS	NS
20 vs. 19	NS	NS	NS	NS
21 vs. 20	NS	NS	NS	NS
22 vs. 21	NS	S- 1.79 (1.21–2.67)	NS	NS
**Drug**	**Tiamulin**	**Tilmicosin**	**Tulathromycin**	**Tildipirosin**
Year	S (p < 0.0001)	S (p < 0.0001)	NS	NS
20 vs. 19	S- 0.06 (0.008–0.46)	S- 0.06 (0.008–0.49)	NS	NS
21 vs. 20	S- 7.75 (2.63–22.8)	S- 4.16 (1.73–9.96)	NS	NS
22 vs. 21	NS	S- 0.28 (0.12–0.68)	NS	NS

S means significant (p < 0.05). OR is significant if the confidence interval 95% does not contain the 1 value

NS means not significant (p > 0.05)

T means statistical tendency

#### Logistic model for Pasteurella multocida

It was not observed any significant temporal trends for susceptibility to quinolones (enrofloxacin and marbofloxacin), amoxicillin, ceftiofur, florfenicol and macrolides (tildipirosin, tulathromycin and tilmicosin) during the study period (p > 0.05). Contrarily, a significant temporal trend (p < 0.05) was observed for tetracyclines (doxycycline and oxytetracycline), tiamulin and sulfamethoxazole/trimethoprim (Table 4).

**Table 4:** The adjusted odds ratio (95% confidence interval) describing the annual variation in susceptibility of *Pasteurella multocida* (PM) isolates to quinolones (enrofloxacin and marbofloxacin), tetracyclines (doxycycline and oxytetracycline), beta-lactams (ceftiofur and amoxicillin), phenicols (florfenicol), sulfonamides (sulfamethoxazol/trimethoprim), pleuromutilins (tiamulin) and macrolides (tilmicosin, tulathromycin and tildipirosin) using the logistic regression model

Drug	Enrofloxacin	Marbofloxacin	Doxycycline	Oxytetracycline
Year	NS	NS	T (p = 0.08)	S (p = 0.018)
20 vs. 19	NS	NS	NS	NS
21 vs. 20	NS	NS	NS	NS
22 vs. 21	NS	NS	NS	S- 2.1 (1.3–3.3)
**Drug**	**Ceftiofur**	**Amoxicillin**	**Florfenicol**	**Sulfamethoxazol/trimethoprim**
Year	NS	NS	NS	S (p = 0.008)
20 vs. 19	NS	NS	NS	S- 4.3 (1.5–11.7)
21 vs. 20	NS	NS	NS	NS
22 vs. 21	NS	NS	NS	NS
**Drug**	**Tiamulin**	**Tilmicosin**	**Tulathromycin**	**Tildipirosin**
Year	S (p = 0.01)	NS	NS	NS
20 vs. 19	NS	NS	NS	NS
21 vs. 20	S- 0.58 (0.34–0.99)	NS	NS	NS
22 vs. 21	S- 2.15 (1.36–3.38)	NS	NS	NS

S means significant (p < 0.05). OR is significant if the confidence interval 95% does not contain the 1 value

NS means not significant (p > 0.05)

T means statistical tendency

**Fig. 2:**
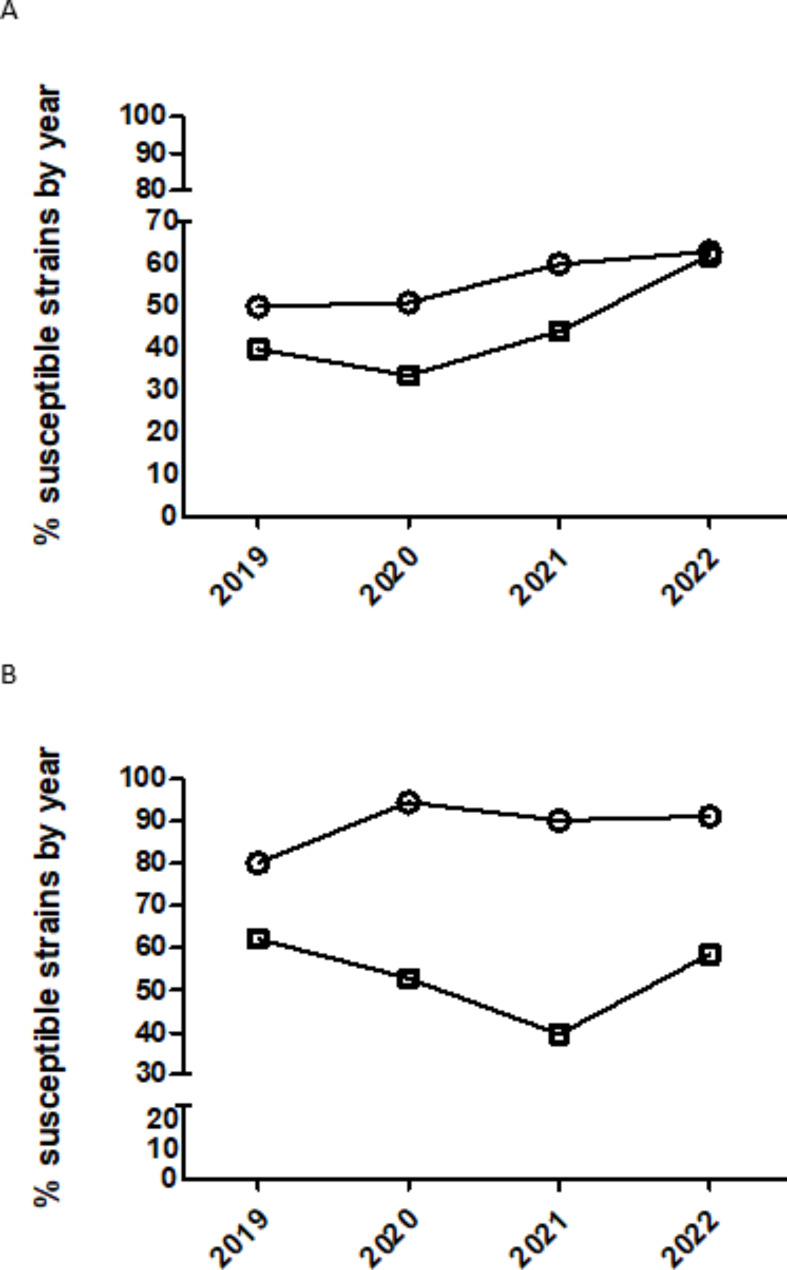
Percentage of susceptible isolates of *Pasteurella multocida* by year for tetracyclines (doxycycline (circle) and oxytetracycline (square) (**A**)) and (sulfamethoxazol/trimethoprim (circle) and tiamulin (square) (**B**)

In the case of tetracyclines, the temporal trend is quite similar for doxycycline (statistical tendency(p < 0.1) for this drug) and oxytetracycline (Table 4; Fig. 2) but only isolates from 2022 had significantly increased odds of being more susceptible than isolates from 2021 for oxytetracycline (Table 4; Fig. 2). For sulfamethoxazole/trimethoprim, isolates from 2020 had significantly increased odds of being more susceptible than isolates from 2019 (Table 4; Fig. 2) remaining without significant changes later. Finally, for tiamulin, isolates from 2021 to 2022 had significantly decreased and increased odds of being more susceptible than isolates from 2020 to 2021, respectively (Table 4; Fig. 2).

## Discussion

Antimicrobial susceptibility is usually measured by the minimum inhibitory concentration (MIC), which is the lowest concentration that stops in vitro growth of the targeted bacteria and using disk diffusion methods in veterinary laboratories. Modelling the MIC values is challenging since these types of data are interval-censored and ordinal [40, 41]. One approach to deal with these data is to dichotomize the MIC values into two categories, resistant (R) and susceptible (S) using established clinical breakpoints or epidemiological cut-off values (ECOFF), followed by logistic regression [42, 43]. Unfortunately, EUCAST ECOFFs are missing for 45.3% (MIC) and 76.9% (disk diffusion) of bacterial species in the veterinary field [37]. Thus, we have decided to carry out our research work using clinical breakpoints instead of ECOFFs. Therefore, we are able to monitor the antimicrobial susceptibility pattern of antibiotics focused on clinical efficacy treatment, but not on monitoring antimicrobial resistance in bacterial populations following the EARS-VET surveillance network proposal [37]. Moreover, our study is based on clinical cases (passive collection) whose representativeness of the general animal population is unknown [44]. Despite these limitations, we consider that our data provide robust information about the antimicrobial susceptibility pattern of the main pig respiratory pathogens in Spain during the study period.

One critical point to dichotomize the MIC values into R and S categories is the existence of accepted clinical breakpoints to obtain comparable results between different studies. In the case of pig respiratory pathogens, there is a reasonable amount of internationally accepted clinical breakpoints [45, 46]. Finally, the antimicrobial panel was selected to represent commonly used compounds for the treatment of pig diseases in practice [13, 14], and not focused on antimicrobial resistance in monitoring programmes. Moreover, comparison of antimicrobial susceptibility from other laboratories must be carried out with caution due to inconsistencies in methodology (MIC versus disk diffusion technique), selection of antimicrobial substances in the test panel and variations in interpretation criteria for clinical breakpoints. We have considered classifying the MIC value obtained for each isolate as susceptible or non-susceptible if there is a high likelihood of therapeutic success or therapeutic failure using a standard posology regimen, respectively. However, for some antimicrobials, it would have been possible to classify the MIC value as susceptible, susceptible at increase antibiotic exposure (former intermediate) and resistant according to latest EUCAST recommendations [47]. However, in the veterinary field, it is very complicated to modify the posology regimen (increase the dose for example) due to the European legislation about antibiotics that recommend following strictly the summary of product characteristics (SPC) [28]. In summary, we believe that our MIC classification of the isolates in susceptible and non-susceptible ones (sum up intermediate and resistant isolates) is closer to the real use of antibiotics under field conditions where the SPC is generally followed by practitioners.

In our study, the isolates of APP were highly susceptible (≥ 85%) for macrolides (tildipirosin, tilmicosin and tulathromycin), tiamulin, florfenicol, sulfamethoxazole/trimethoprim and ceftiofur. However, the antimicrobial susceptibility was intermediate (41–85%) for amoxicillin, quinolones (enrofloxacin and marbofloxacin) and low (0–40%) for doxycycline and oxytetracycline. This antimicrobial susceptibility pattern described for APP in this study agrees with results obtained by Spanish researchers with isolates collected from 1997 for florfenicol and amoxicillin [48]. Moreover, our results are quite similar for isolates from other European countries with some differences [49] in some antimicrobial families such as tetracyclines (70.1% in Europe versus 8.9% in our study (lowest observed value)), quinolones (97.6% in Europe versus 69.9% in our study (lowest observed value)), amoxicillin (94.7% in Europe versus 62.1% in our study (lowest observed value)) and tilmicosin (80.5% in Europe versus 88.7% in our study (lowest observed value)). It must be highlighted the extreme difference observed for tetracyclines between most European countries and Spain. Overall, there are still good opportunities to treat infections by APP with antimicrobials, but the presence of isolates resistant to tetracyclines (doxycycline and oxytetracycline), amoxicillin and quinolones (enrofloxacin and marbofloxacin) in Spain highlights the importance of monitoring antimicrobial susceptibility and select the most suitable antimicrobial in a case-by-case situation [14]. On the other hand, the isolates of PM were highly susceptible (≥ 85%) to macrolides (tildipirosin, tilmicosin and tulathromycin), florfenicol, quinolones, amoxicillin and ceftiofur. However, the antimicrobial susceptibility was intermediate (41–85%) for sulfamethoxazole/trimethoprim, tiamulin and low (0–40%) for doxycycline and oxytetracycline. Moreover, the antimicrobial susceptibility pattern described in our study is similar to the pattern described by Spanish researchers with isolates collected from 1987 [50] and by European researchers in multi-country studies to determine the antimicrobial susceptibility of PM in pigs [49, 51] with clear differences in tetracyclines (74.1% in Europe versus 33.3% in our study (lowest observed value)), and slight differences for sulfonamides (94.1% in Europe versus 80.2% in our study (lowest observed value)) and tiamulin (59.1% in Europe versus 39.6% in our study (lowest observed value)). Again, it must be highlighted the extreme difference in antimicrobial susceptibility observed for tetracyclines between most European countries and Spain. Globally, the antimicrobial susceptibility of PM seems that have not changed significantly across time for most of the studied antimicrobial families, at least, in Europe as recently published [49] comparing data from 2009 to 2012 to 2015–2016. It cannot be ruled out that pig movements among farms and European countries may have an impact on antibiotic resistance in these bacteria at population level. Thus, some respiratory pathogen strains could be shared between European countries with pig commercial relationships such as Spain with Denmark, Holland, and Germany [52].

In general terms, pig pathogens (APP and PM) involved in respiratory diseases analysed herein appeared to remain susceptible or tended to increase susceptibility to antimicrobials over the study period (2019–2022) but our data clearly showed a different pattern in the evolution of antimicrobial susceptibility for each combination of drug and microorganism. Thus, there are combinations of bacteria and antimicrobial with no significant changes in their susceptibility pattern for antimicrobials during the last four years such as APP and PM for cephalosporins, phenicols and macrolides. For these drugs, it must be highlighted that there is a limitation to observe a trend in the population because the proportion of non-susceptible isolates is very low in the study population. On the other hand, there are pairs of drug and microorganism with significant changes such as APP and quinolones, tetracyclines, amoxicillin, pleuromutilins and tilmicosin and PM and tetracyclines, pleuromutilins and sulfonamides. This result is very interesting because both bacteria are in the same ecological niche and reinforced that the evolution of antimicrobial susceptibility must be studied in a case-by-case situation where generalization for drug families and bacteria is not possible as described previously by our research group [14]. One interesting line of research could be studying the evolution mechanisms shaping the maintenance of antibiotic resistance in pig respiratory pathogens as carried out by Durao et al. [53] but it is out of the scope of this paper.

One hallmark of the European legislation in relation with AB is to decrease the use of these drugs in livestock with the goal of decreasing the AMR burden not only in animals but also in humans. Following this rationale, a national program to combat antimicrobial resistance (https://resistenciaantibioticos.es/es) has been developed and carried out since 2014 in Spain. As a consequence, the antimicrobial consumption in livestock has been reduced 62.4% from 2014 to 2021 in Spain [54]. It would have been ideal to carry out a study to link this AB consumption in pigs with the antimicrobial susceptibility trend observed. Unfortunately, it was not feasible because AB consumption is not available at farm level. Our idea is to collect this information at farm level to analyse the data about antimicrobial use with a multivariable model, including the way antimicrobials are used on the farm, routes of administration, duration of antimicrobial use, veterinary control, herd size, and the level of biosecurity and sanitation [55, 56] following a similar methodology by other researchers focused on human health [22].

## Methods

### Clinical samples

Between January 2019 and December 2022, samples were taken from diseased or recently deceased pigs from farms across Spain showing acute clinical signs of respiratory tract infections. Only one isolate was included by farm across the study to avoid redundancy and overrepresentation of bacterial clones. None of these animals had been exposed to antimicrobial treatment for, at least, 15 days prior sampling. Thus, the sampled animals were between 3 and 24 weeks old showing overt respiratory symptoms with or without depression and/or hyperthermia (> 39.8ºC). For each clinical case, samples of lungs of two recently deceased pigs (< 12 h) were submitted under refrigeration to the laboratory. If no recently dead pigs were suitable for sampling, at least, two animals with acute respiratory signs were humanely sacrificed and lung samples (whole lung or the lung lobule/s with overt lesions) were drawn by the clinician with diagnostic purposes. A swab was drawn from the lung lesions after sterilization of its surface to avoid bacterial surface contamination.

### Bacterial isolation and identification

Clinical specimens were cultured aseptically onto blood agar (Columbia agar with 5% Sheep blood, 254,005 BD), chocolate agar (GC II agar with IsoVitaleX, 254,060, BD or blood Agar No. 2 Base, 257,011, BD) and MacConkey agar (4,016,702, Biolife Italiana Srl) and incubated at 35–37 °C in aerobic conditions with 5–10% CO_2_ for 24–48 h to address the isolation of respiratory bacterial pathogens.

Identification of isolates for respiratory pathogens was carried out by matrix assisted laser desorption ionization-time of flight (MALDI-TOF Biotyper System, Bruker Daltonics, Bremen, Germany) as previously described [13]. *Actinobacillus pleuropneumoniae* was also confirmed by PCR technique due to limitation of MALDI-TOF for the *Actinobacillus* genus [57]. Individual isolates were stored at -80 °C in brain heart infusion (CM1135, Oxoid) with 30% of glycerol (G9012, Sigma-Aldrich).

### Antimicrobial susceptibility testing

Antimicrobial susceptibility testing was determined by microdilution test to obtain the minimum inhibitory concentration (MIC) value for each combination of bacterial species and antimicrobial tested. Thus, MIC was performed in accordance with the recommendations presented by the Clinical and Laboratory Standards Institute [45, 46] in a customized 96-well microtitre plate (Sensititre, Trek diagnostic Systems Inc., East Grinstead, UK) containing a total of 12 and 8 antibiotics/concentrations for respiratory pathogens respectively. The antimicrobials and the range of concentrations tested for swine respiratory pathogens belong to category D [13, 14]: Sulfamethoxazole/trimethoprim (0.06-4 µg/mL), doxycycline (0.12-16 µg/mL), oxytetracycline (0.12-16 µg/mL) and amoxicillin (0.06-8 µg/mL); Category C: Florfenicol (0.06-8 µg/mL), tiamulin (0.5–64 µg/mL), tulathromycin (0.5–64 µg/mL), tildipirosin (0.5–64 µg/mL) and tilmicosin (0.5–64 µg/mL) and category B: Ceftiofur (0.03-4 µg/mL), enrofloxacin (0.03-4 µg/mL) and marbofloxacin (0.03-4 µg/mL).

Bacteria were thawed, cultured on chocolate agar or blood agar, and incubated at 35–37ºC in aerobiosis (or with 5–10% CO_2_ for APP) for 18-24 h. Three to five colonies were picked and emulsified in demineralized water (or Cation Adjusted Muëller-Hinton Broth (CAMHB) for APP) to obtain a turbidity of 0.5 McFarland standard (Sensititre™ nephelometer V3011). Suspensions were further diluted in CAMHB with 2.5-5% Lysed Horse Blood for PM and Veterinary Fastidious Medium (VFM) or Mueller Hinton Fastidious broth with Yeast (MHF-Y) for APP to reach a final inoculum concentration of 5 × 10^5^ cfu/ml. Then, the Sensititre panel was reconstituted by adding 100 µl/well of the inoculum. PM isolates were incubated at 35 ± 2ºC for 18-24 h. In the case of APP isolates, plates were covered with a perforated seal and incubated at 35 ± 2ºC with 5–10% CO2 for 20-24 h.

The antibiotic panels were read manually using Sensititre™ Vizion (V2021) and the MIC value was established as the lowest drug concentration inhibiting visible growth. For each isolate tested, a colony count and a purity check were performed following CLSI and manufacturer recommendations. Moreover, quality control isolates were also included. Thus, *Actinobacillus pleuropneumoniae* (ATCC 27,090™), *Escherichia coli* (ATCC 25,922™), *Streptococcus pneumoniae* (ATCC 49,619™) and *Enterococcus faecalis* (ATCC 29,212™) were included as quality control following CLSI recommendations [45, 46]. The MICs of the quality control isolates had to be within acceptable CLSI ranges to accept the results obtained in the laboratory.

### Statistical methods

All the data analysis was carried out with JMP®, Version 13 (SAS Institute Inc., Cary, NC, USA, 1989–2019). Descriptive statistics (MIC range, MIC_50_ and MIC_90_) were performed to summarize the distribution of the isolates within each MIC category. Clinical susceptibility (susceptible/non-susceptible for each isolate) was determined according to CLSI clinical breakpoints for APP and PM (supplemmentary Table 1). If the MIC value observed was lower (or equal) or higher than the clinical breakpoint was classified as susceptible or non-susceptible, respectively for each antimicrobial. Thus, the non-susceptible category include intermediate and resistant isolates according to CLSI recommendations [45]. Moreover, the percentage of susceptible isolates for each combination of antimicrobial and microorganism is classified as low (0–40%), intermediate (41–85%) and high (> 85%) to make easier the description of the results.

A logistic (susceptible/resistant for each isolate) was used to analyse the susceptibility data for the antimicrobials from year 2019 to 2022, only for those pairs of antimicrobial/microorganisms if at least 100 isolates were available for each year, as recommended by De Jong et al. (2022) [34]. Susceptible/resistant data were used for logistic regression model as dependent variables, and the year as independent one. Thus, year of sampling was categorized by individual years and modelled as a hierarchical indicator variable, where for each year the preceding year was used as the referent [58]. The model assumptions and goodness-of-fit were evaluated as appropriate for these models [58]. Thus, the level of significance used to reject the null hypothesis was p ≤ 0.05.

### Electronic supplementary material

Below is the link to the electronic supplementary material.

**Supplementary table 1**: Clinical breakpoints (susceptible/non-susceptible for each isolate) were used according to Clinical Laboratory Standards institute (CLSI) recommendations for Actinobacillus pleuropneumoniae (APP) and Pasteurella multocida (PM). The non-susceptible category includes intermediate and resistant isolates according to CLSI recommendations.

## Data Availability

The data supporting the conclusions of this article are included within the article and in the additional file. Moreover, additional datasets are available upon reasonable request.

## Declarations

Not applicable as only clinical cases were included for diagnostic purposes.
