# Supplementary material for: Susceptibility trends of swine respiratory pathogens from 2019 to 2022 to antimicrobials commonly used in Spain
Source: Porcine Health Manag. 2023 Oct 19;9:47. doi: 10.1186/s40813-023-00341-x (PMC10588200; doi:10.1186/s40813-023-00341-x)
Supplement: Supplementary file 1 — Supplementary table 1: Clinical breakpoints (susceptible/non-susceptible for each isolate) were used according to Clinical Laboratory Standards institute (CLSI) recommendations for Actinobacillus pleuropneumoniae (APP) and Pasteurella multocida (PM). The non-susceptible category includes intermediate and resistant isolates according to CLSI recommendations. [file 40813_2023_341_MOESM1_ESM.docx]

Supplementary table 1

Clinical breakpoints (susceptible/non-susceptible for each isolate) were used according to Clinical Laboratory Standards institute (CLSI) recommendations for *Actinobacillus pleuropneumoniae* (APP) and *Pasteurella multocida* (PM). The non-susceptible category includes intermediate and resistant isolates according to CLSI recommendations.

| **Antimicrobial** | **APP** | | **PM** | |
| --- | --- | --- | --- | --- |
|  | Susceptible | Non-susceptible | Susceptible | Non-susceptible |
| Amoxicillin^1^ | <0.5 | >0.5 | <0.5 | >0.5 |
| Ceftiofur | <2 | >2 | <2 | >2 |
| Doxycycline^2^ | <0.5 | >0.5 | <0.5 | >0.5 |
| Enrofloxacin | <0.25 | >0.25 | <0.25 | >0.25 |
| Florfenicol | <2 | >2 | <2 | >2 |
| Marbofloxacin^3^ | <0.25 | >0.25 | <0.25 | >0.25 |
| Oxytetracycline^2^ | <0.5 | >0.5 | <0.5 | >0.5 |
| Sulfamethoxazol/trimethoprim^4^ | <2 | >2 | <2 | >2 |
| Tiamulin | <16 | >16 | <16 | >16 |
| Tilmicosin | <16 | >16 | <16 | >16 |
| Tildipirosin | <16 | >16 | <4 | >4 |
| Tulathromycin | <64 | >64 | <16 | >16 |

All clinical breakpoints were obtained from CLSI. Performance Standards for Antimicrobial Disk and Dilution Susceptibility Tests for Bacteria Isolated from Animals. 5th ed. CLSI supplement VET01S. Wayne, PA: Clinical laboratory institute; 2021, with the following clarifications: ^1^ Schwarz et al. (2008) and it has been also extrapolated from ampicillin. ^2^ Extrapolated from tetracycline. ^3^ Extrapolated from enrofloxacin. ^4^ MIC represented in the table is for trimethoprim. Sulfamethoxazol/trimetropim ratio tested is 19:1.

Additional bibliography:

Schwarz S, Böttner A, Goossens L, Hafez HM, Hartmann K, Kaske M, Kehrenberg C, Kietzmann M, Klarmann D, Klein G, Krabisch P, Luhofer G, Richter A, Schulz B, Sigge C, Waldmann KH, Wallmann J, Werckenthin C. A proposal of clinical breakpoints for amoxicillin applicable to porcine respiratory tract pathogens. Vet Microbiol. 2008;126(1-3):178-88.
